# 肺部转移性肿瘤患者的手术方式选择及预后因素分析

**DOI:** 10.3779/j.issn.1009-3419.2015.04.06

**Published:** 2015-04-20

**Authors:** 烨 徐, 连伟 白, 良 张, 锋 毛, 屠阳 申

**Affiliations:** 1 200336 上海，上海交通大学医学院附属同仁医院胸外科 Department of Toracic Surgery, Tongren Hospital, Shanghai Jiaotong University, School of Medicine, Shanghai 200336, China; 2 130012 长春，吉林省肿瘤医院 Jilin Tumor Hospital, Changchun 130012, China; 3 200030 上海，上海交通大学附属胸科医院 Shanghai Chest Hospital, Shanghai Jiaotong University, Shanghai 200030, China

**Keywords:** 肺转移瘤, 外科治疗, 预后因素, Pulmonary metastases, Surgical treatment, Prognostic factors

## Abstract

**背景与目的:**

肺部转移性肿瘤的治疗观念有所改变，外科手术的治疗价值及影响预后的因素值得探讨。

**方法:**

回顾性分析2006年1月-2009年12月经手术治疗的57例肺部转移性肿瘤患者的临床资料，随访1年、3年、5年生存率，比较胸腔镜和常规开胸两种手术方式的差别，探讨患者性别、年龄、手术方式、无瘤生存间隔期（disease-free interval, DFI）、转移瘤数目、转移瘤大小及术后是否化疗与预后的关系。

**结果:**

全组患者围手术期无死亡，患者术后1年、3年、5年生存率分别为81.3%、46.5%、29.2%，中位生存时间为33.8个月。多因素分析显示DFI、转移瘤数目和直径是影响预后的独立因素。

**结论:**

合理选择手术治疗，能够提高肺部转移性肿瘤患者的生存期，胸腔镜是优选的手术方式，孤立性肺转移瘤及直径 < 4 cm的患者手术效果更佳。

## Abstract (en)

**Background and objective:**

Concept of treatment for pulmonary metastatic tumor has been changed. The aim of this study is to analysis of the choice of operation mode and prognosis factors of patients with tumors of the lung metastasis.

**Methods:**

The clinical data of 57 patients with pulmonary metastases who underwent operations from January 2006 to December 2009 were retrospectively analyzed. Difference of conventional open thoracic surgery and thoracoscope surgery was compared. The relationship between gender, age, type of surgery, disease-free interval (DFI), the number of metastatic tumor, the size of tumor, chemotherapy and prognosis was explored.

**Results:**

Among the patients, there was no perioperative mortality. Postoperative 1-, 3-, 5-year survival rates were 81.3%, 46.5% and 29.2%, the median survival time was 33.8 months. Multivariate analysis showed that DFI, the number and diameter of metastasis tumor were independent prognostic factors.

**Conclusions:**

Reasonable choice of surgical treatment can improve the survival of pulmonary metastatic tumor patient. Thoracoscopic surgery method is preferred. Operation effect of patients with tumor diameter less than 4 cm in solitary pulmonary metastasis has better effect.

肺是恶性肿瘤最常见的转移靶器官之一，晚期恶性肿瘤的肺转移率高达40%-50%，其中在1/5患者中肺是唯一的转移部位^[1, 2]^。通常认为恶性肿瘤出现远处转移时，转移靶器官局部手术的价值不高，对提升患者生存率的贡献不大。近年，国外文献^[3-6]^报道，部分肺转移瘤患者可从相关肺手术中获益，肺转移瘤的外科治疗往往亦是影响患者生存的重要因素。但迄今为止，罕有肺转移瘤外科手术与姑息治疗的前瞻性随机对照临床试验，本文拟回顾性分析两种手术治疗患者的相关数据，希望为肺转移瘤外科治疗的选择指证和预后价值提供临床依据。

## 1 资料和方法

### 1.1 一般资料

选择2006年1月-2009年12月上海市同仁医院胸外科、吉林省肿瘤医院肿瘤内科及上海市胸科医院肺部肿瘤临床医学中心收治的手术治疗肺转移瘤患者中有完整随访记录患者共57例。年龄16岁-75岁，中位年龄54岁，其中男性30例，女性27例。患者在原发肿瘤常规复查时发现肺转移瘤28例，其余29例无明确其他部位原发肿瘤史。患者主要症状包括：刺激性咳嗽、咳痰（15例），痰血（5例），胸闷、气短（3例），胸背部疼痛（2例），其他症状包括发热、乏力（4例）。患者入院常规行肺功能、心电图、血常规、凝血功能、肝肾功能等检查确定心肺功能及一般情况，评估能够耐受手术，无其他影响手术的伴发疾病。所有患者术前均行胸部计算机断层扫描（computed tomography, CT）检查明确肺部病灶可完全切除。骨发射型CT（emission CT, ECT）、头颅磁共振成像（magnetic resonance imaging, MRI）/CT、腹部CT或正电子发射型计算机断层显像（positron emission computed tomography, PET）-CT检查均未发现肺外转移。

### 1.2 原发肿瘤的病理类型

详见表 1。

**1:** 原发肿瘤的病理类型 Primary tumor pathological type

Pathology	Cases	Percentage (%)
Breast cancer	6	10.53
Colorectal cancer	15	26.32
Kidney cancer	6	10.53
Thyroid cancer	3	5.26
Sarcoma	9	15.79
Germ cell tumors	7	12.28
Malignant melanoma	1	1.75
Cervical cancer	4	7.02
Endometrial cancer	5	8.77
Brain cancer	1	1.75
Total	57	100.00

### 1.3 转移瘤的数目及大小

全组单发肺转移瘤32例，2个以上多发转移瘤25例，其中同一肺叶内12例，同侧肺不同肺叶13例。肿瘤直径≤4 cm者42例，直径 > 4 cm者15例。

### 1.4 无瘤生存间隔期（disease-free interval, DFI）

即原发肿瘤手术后至肺转移瘤出现的间隔时间，本组4个月≤DFI≤78个月，中位26.8个月，≤36个月者38例（66.7%）， > 36个月者19例（33.3%）。几种常见的肺转移瘤病理类型乳腺癌DFI最长为43.1个月，结直肠癌31.7个月，肉瘤18.3个月，肾癌24.9个月。

### 1.5 治疗方法

#### 1.5.1 手术方法

全组患者均采用双腔气管插管麻醉，行肺转移瘤切除手术。手术根据转移瘤部位、数目、大小采用后胸部后外侧切口或胸腔镜手术，其中常规开胸手术21例，胸腔镜手术36例。手术方式包括：肺楔形切除术43例、肺段切除术3例、肺叶切除术11例。图 1A为左侧乳腺癌术后患者，常规复查胸部CT提示左下肺结节，考虑为转移性，行电视辅助胸腔镜手术（video-assisted thoracic surgery, VATS）左肺下叶结节楔形切除术，术后病理（图 1B）证实为转移性乳腺癌。图 1C为直肠癌切除术后患者，因咳嗽症状行胸部CT提示右肺下叶结节，考虑为转移性，常规开胸行右肺下叶切除术，术后病理（图 1D）证实为腺癌，考虑胃肠道来源。

**1:**
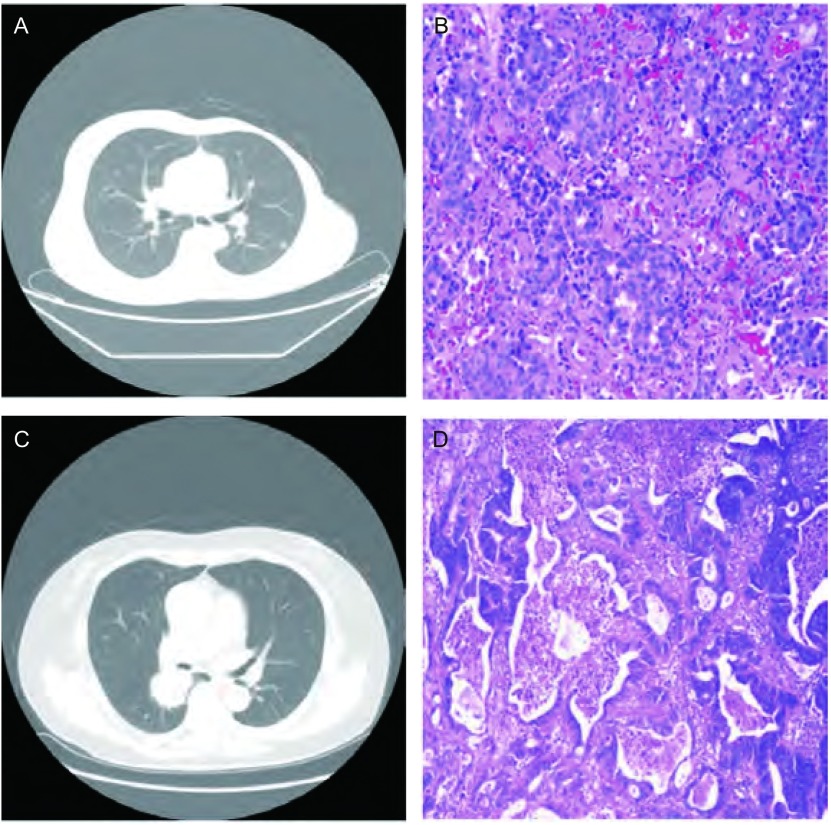
代表性图片。A：左下肺结节；B：转移性腺癌（HE染色，×200）；C：右肺下叶结节；D：转移性腺癌（HE染色，×200）。 Representative pictures. A: Left lower lobe nodules; B: Metastatic adenocarcinoma (Hematoxylin-eosin staining, ×200); C: Right lower lobe nodules; D: Metastatic adenocarcinoma (Hematoxylin-eosin staining, ×200).

#### 1.5.2 辅助治疗

全组术后行化疗者24例（42.11%），未化疗者33例（57.89%）。化疗根据原发肿瘤病理类型选择不同方案。未化疗者多为原发肿瘤对化疗不敏感性肿瘤，包括肾癌、甲状腺癌、肉瘤、黑色素瘤等。

### 1.6 统计学方法

采用SPSS 13.0统计学软件处理，组间比较采用*t*检验或卡方检验，生存率及预后单因素分析采用*Kaplan*-*Meier*法，*Cox*回归比例风险模型进行多因素预后分析，*P* < 0.05为差异有统计学意义。

## 2 结果

### 2.1 手术方式

VATS手术并发症3例，包括2例肺漏气、皮下气肿，1例术后心律失常；常规开胸手术并发症6例，包括2例术后心律失常，1例肺部感染，1例肺不张，1例术后胸腔出血，1例肺漏气。两组均无手术死亡的患者。由表 2可知两组手术方式胸腔镜组并发症更少，但5年生存率没有统计学差异。此外，VATS组的术中失血量、术后引流量及住院时间均要少于常规开胸组，但VATS组手术时间要长于常规开胸组。

**2:** 比较常规开胸与胸腔镜手术方式 Comparison of difference of conventional open thoracic surgery and thoracoscope surgery

Item	VATS	Thoracotomy	*P*
Complications	3/36	6/21	< 0.05
Survival rate (5-year)	27.78%	33.33%	> 0.05
Blood loss (mL)	118.1±34.7	205.1±49.3	< 0.05
Postoperative drainage (mL)	1078.4±215.6	1564.3±387.9	< 0.05
Hospital stay (d)	6.3±1.8	7.8±1.5	< 0.05
Operation time (min)	129.1±38.3	102.1±40.2	< 0.05
VATS: video-assisted thoracic surgery.			

### 2.2 预后因素分析

#### 2.2.1 全组患者生存率分析

患者术后1年、3年、5年生存率分别为81.3%、46.5%、29.2%，中位生存时间为33.8个月。

#### 2.2.2 单因素分析

全组病例对性别、年龄、手术方式、DFI、转移瘤数目、单发转移瘤大小及术后是否化疗进行单因素分析，结果显示：性别、手术方式、术后是否化疗均不是肺转移瘤切除术后影响生存率的因素（*P* > 0.05）。年龄 > 60岁、DFI > 36个月、单发转移瘤、转移瘤直径≤4 cm的患者5年生存率更高，差异有统计学意义（*P* < 0.05）（表 3）。

**3:** 肺转移瘤患者预后的单因素分析 Single factor analysis of the prognosis of patients with lung cancer metastasis

	*n*	5-year survival rate (%)	*P*
Gender			0.391
Male	30	28.7	
Female	27	31.3	
Age (yr)			0.004
≤60	36	21.9	
> 60	21	36.5	
DFI (mo)			< 0.001
≤36	38	19.8	
> 36	19	40.2	
Surgery			0.412
Partial resection	46	27.9	
Lobectomy	11	30.8	
Numbers of tumor			0.026
1	32	34.8	
≥2	25	21.6	
Size of tumor (cm)			< 0.001
≤4	42	37.2	
> 4	15	11.9	
Chemotherapy			0.403
Yes	24	30.8	
No	33	27.6	
DFI: disease-free interval.			

#### 2.2.3 多因素分析

将年龄、DFI、转移瘤数目、转移瘤直径带入Cox多因素回归分析，结果显示DFI、转移瘤数目、转移瘤直径是影响肺转移瘤切除术后预后的独立因素（表 4）。

**4:** 肺转移瘤患者预后的多因素分析 Multivariate analysis of prognostic factors in the patients with lung metastasis

	*P*	RR	95%CI	
			Lower	Upper
Age	0.059	1.779	0.978	3.238
DFI	0.021	1.909	1.101	3.308
Size of tumor	0.042	0.514	0.271	0.975
Numbers of tumor	0.035	0.713	0.264	0.857

## 3 讨论

近年来，国内外许多学者注意到了外科手段治疗肺转移瘤的潜在优势，并通过对大宗病例的总结和文献报道，显示外科手术作为肺转移瘤的一种治疗手段，可为患者提供长期生存的机会。对于肺转移瘤外科治疗报道的文献^[7-9]^较多，大都取得了良好的效果。本研究组总体5年生存率达29.2%，而国外研究^[10]^发现原发肿瘤控制后肺转移瘤采取非手术治疗5年生存率不足10%，外科手术的介入提高了这部分患者的远期生存率。外科治疗肺转移瘤的可行性主要基于以下几点：①肺脏是许多恶性肿瘤最常见和最初转移的部位，手术切除可阻止其进一步扩散；②肺脏也可能是某些恶性肿瘤唯一转移的器官，这部分患者如果采取积极的手术干预或许会取得良好的效果；③部分肿瘤对放化疗敏感性较差，如骨肉瘤、软组织肉瘤、肾透明细胞癌、黑色素瘤、甲状腺癌等，故手术成为首选；④目前大宗文献报道肺转移瘤的手术效果较好且死亡率较低。

虽然文献报道外科治疗肺转移瘤取得了良好效果，然而真正符合手术标准的病例仅占所有肺转移瘤患者的极少数，因而病例的选择至关重要。以往人们认为恶性肿瘤有了远处转移即为手术禁忌，然而有学者^[11, 12]^对这些“禁区”进行了尝试，首例肺转移瘤切除1927年由Divis在欧洲报道，美国Barney和Churchill在1939年对肾癌肺转移的患者进行了肺转移瘤的手术切除，结果患者在术后存活了23年。目前，一系列回顾性研究^[5, 6]^表明，对符合适应证的肺转移瘤患者积极进行手术治疗，可使生存期延长。Rusch等^[12]^1995年提出了肺转移癌手术切除病例选择标准：①原发肿瘤得到局部控制；②无胸外其他部位转移灶；③肺部转移灶能够完全切除；④无其他有效的系统治疗方法；⑤能耐受癌肿切除术。我们认为，除上述几点，还应包括目前多数学者主张的选择标准：①胸部影像学检查等证据表明符合肺转移瘤的诊断；②肺转移癌病灶在4个及以下并位于同一肺叶内或同侧胸腔内；③病理证实原发瘤的恶性程度低，手术切除效果好；④原发瘤经治疗后有较长的无瘤时间。对于原发瘤治疗后较早出现的肺转移癌，因可能很快会出现肺以外其他部位转移灶，手术应慎重考虑。此外，病例的选择在个别情况下也可以有一定的延伸和改进，有学者认为肺转移瘤同时或异时伴有其他脏器的转移灶在能够切除的情况下不应该作为手术禁忌，积极的手术切除同样能获得良好的生存率，特别是对于伴有孤立性肝转移灶的结直肠癌肺转移瘤患者，手术仍是可选择的治疗方法。Joosten等^[13]^报道的39例手术切除的结直肠癌肺转移瘤患者中，21例伴有肝转移及肺转移患者，分期切除后5年生存率仍达到20%。有研究^[14]^报道58例结直肠癌术后同时或异时行肝转移瘤切除术的患者，5年及10年生存率分别为30%及16%，中位生存期为62个月。Daniela等^[15]^也认为对部分心肺功能好，肿瘤能够完全切除的患者反复手术是可行的，其报道的330例手术患者中，35例行2次或多次肺转移瘤切除术，2次术后平均生存期为26.5个月。在没有其他有效治疗手段情况下，如肿瘤对放化疗不敏感，对肺转移瘤手术切除应持积极态度。因此，合适病例的选择应该结合患者本身条件、原发肿瘤及肺转移瘤三方面因素综合考虑，目的是为了最大限度的提高患者远期生存率及生活质量。

肺转移瘤的手术方式选择趋向于保守，提倡经济切除，即在转移瘤能够完全切除的前提下，应该尽可能的保留患者正常肺组织，提高术后生活质量。术式的选择应该根据转移瘤的大小、部位、侵犯范围而定，肺部分切除仍然是主要术式，包括肺楔形切除和肺段切除。在肺部分切除无法达到完全切除的情况下，肺叶切除也值得推荐，但应尽可能避免全肺切除。本研究也发现，肺叶切除仍然占有一定比例，虽然创伤比部分肺切除稍大，但并没有因此而影响患者远期生存率。随着胸腔镜技术的发展和推广应用，许多学者开始采用VATS切除肺转移瘤，其优点在于创伤小，术后恢复快，术后患者生活质量高。

国际肺转移瘤登记组织（International Registry of Lung Metastases, IRLM）^[7]^对欧美18个中心共5, 206例肺转移瘤切除的回顾性分析表明，生存率最重要的决定因素是转移瘤的可切除性：完全切除者5年生存率为36%，不完全切除者仅为13%；完全切除的病例中有53%出现再发，再次接受转移癌切除者预后优于未再手术者；Koong等^[16]^对全肺切除治疗肺转移瘤进行的研究发现，133例第一次治疗肺转移瘤即采用全肺切除的患者，根治组5年生存率达20%，而不完全切除生存未超过2年者，肺转移瘤术后再次复发而行全肺切除的患者中，5年生存率达30%，未达到根治者5年生存率为0，因而认为长期生存主要取决于能否完全切除转移灶。关于切除范围，Pfannschmidt等^[17]^系统性回顾了20个结肠癌肺转移的研究，发现切除范围不是生存时间的影响因素，支持进行有限的肺切除，在切缘阴性的同时尽量保存肺功能，对肺转移瘤不适宜做全肺切除^[18]^。而IRLM的5, 206例资料中，行楔形切除、肺段切除、肺叶或双肺叶切除、全肺切除的比例分别是67%、9%、21%和3%，楔形切除可达到与解剖性切除相同的效果。

DFI即原发肿瘤手术后至肺转移瘤出现的时间，本研究对DFI≤36个月及 > 36个月两组进行单因素及多因素分析发现，DFI是肺转移瘤切除术后影响预后的独立因素，DFI > 36个月患者5年生存率为40.2%，而DFI≤36个月患者仅为19.8%。国外多数报道^[19, 20]^也认为DFI是影响预后的重要因素。其理论基础可能是DFI越长，其肿瘤倍增时间也越长。因此，DFI反映了肿瘤的进展情况，DFI越短，肿瘤进展越快，反之DFI越长，肿瘤进展越慢，患者也就相应有更长的生存期。不过DFI并不作为病例选择的限制条件，即使同期发现原发灶及转移瘤，手术也是可行的。

关于转移肿瘤的个数对术后生存的影响，Takita等^[21]^报道一组病例的中位生存期，单个转移者为27.3个月，多个转移者仅为17个月，二者之间差异有统计学意义。本组单个转移瘤患者术后5年生存率为34.8%；多个转移瘤5年生存率为21.6%。有研究^[22]^认为，手术对2个以上的转移灶益处不大。我们认为对肺转移瘤个数超过4个者手术应慎重。

结合本研究和相关文献，我们认为：合理选择患者，完全切除转移灶，可以延长部分肺转移瘤患者的生存期。在转移瘤完全切除的前提下，应尽可能保留患者肺功能，胸腔镜是优选的手术方式。对DFI时间长、孤立性肺转移瘤及直径≤4 cm肺转移瘤的手术效果更佳。

## References

[1] Nichols FC (2012). Pulmonary metastasectomy. Thorac Surg Clin.

[2] Hornbech K, Ravn J, Steinbrüchel DA (2011). Current status of pulmonary metastasectomy. Eur J Cardiothorac Surg.

[3] Chen F, Fujinaga T, Sato K (2009). Clinical features of surgical resection for pulmonary metastasis from breast cancer. Eur J Surg Oncol.

[4] Ludwig C, Stoelben E, Hasse J (2003). Disease-free survival after resection of lung metastases in patients with breast cancer. Eur J Surgical Oncol.

[5] Pfannschmidt J, Egerer G, Bischof M (2012). Surgical intervention for pulmonary metastases. Dtsch Arztebl Int.

[6] Corona-Cruz JF, Domínguez-Parra LM, Saavedra-Pérez D (2012). Lung metastasectomy: Long-term outcomes in an 18-year cohort from a single center. Surg Oncol.

[7] Pastorino U, Buyse M, Friedel G (1997). Long-term results of lung metastasectomy: prognostic analyses based on 5, 206 cases. J Thorac Cardiovasc Surg.

[8] Welter S, Jacobs J, Krbek T (2007). Long-term survival after repeated resection of pulmonary metastases from colorectal cancer. Ann Thorac Surg.

[9] Rena O, Papalia E, Oliaro A (2006). Pulmonay metastases from epithelial tumours: late results of surgical treatment. Eur J Cardiothorac Surg.

[10] Headrick JR, Miller DL, Nagomery DM (2001). Surgical treatment of hepatic and pulmonary metastases from colon cancer. Ann Thorac Surg.

[11] Fujimura S, Kondo T, Yamauchi A (1984). A ten-year experience with surgical resection for patients with metastatic lung tumors. Tohoku J Exp Med.

[12] Rusch VW (1995). Pulmonary metastasectomy. Current indications. Chest.

[13] Joosten J, Bertholet J, Keemers-Gels M (2008). Pulmonary resection of colorectal metastases in patients with or without a history of hepatic metastases. J Cancer Surg.

[14] Headrick JR, Miller DL, Nagorney DM (2001). Surgical treatment of hepatic and pulmonary metastases from colon cancer. Ann Thorac Surg.

[15] Daniela K, Elisabeth K, Heinz T (1998). Long-term results after repeated surgical removal of pulmonary metastases. Ann Thorac Surg.

[16] Koong HN, Pastorino U, Ginsberg RJ (1999). Is there a role for pneumonectomy in pulmonary metastases? International Registry of Lung Metastases. Ann Thorac Surg.

[17] Pfannschmidt J, Dienemann H, Hoffmann H (2007). Surgical resection of pulmonary metastases from colorectal cancer: a systematic review of published series. Ann Thorac Surg.

[18] Migliore M, Jakovic R, Hensens A (2010). Review extending surgery for pulmonary metastasectomy: what are the limits?. J Thorac Oncol.

[19] Rena O, Papalia E, Oliaro A (2006). Pulmonary metastases from epithelial tumours: late results of surgical treatment. Eur J Cardiothorac Surg.

[20] Zabaleta J, Aguinagalde B, Fuentes MG (2011). Review and update of the prognostic factors in lung metastasis surgery. Cir Esp.

[21] Takita H, Edgerston F, Karakousis M (1991). Surgical managment of metastases to the lungs. Surg Gynecol Obstet.

[22] Mansel JK, Zinsmicister AR, Pairolero PC (1996). Pulmonary resection of metastatic colorectal adenocarcinoma. Chest.

